# Genetic Variability and Association of Morpho-Agronomic Traits Among Ethiopian Barley (*Hordeum vulgare* L) Accessions

**DOI:** 10.1155/sci5/3957883

**Published:** 2025-02-07

**Authors:** Alemayehu Zewodu, Wassu Mohammed, Eleni Shiferaw

**Affiliations:** ^1^Department of Crop and Horticulture Biodiversity Research, Ethiopian Biodiversity Institute, Addis Ababa, Ethiopia; ^2^Department of Plant Science, Haramaya University, Dire Dawa, Ethiopia

**Keywords:** barley, cluster, correlation, Euclidian distances, genetic advance, heritability, principal components

## Abstract

Barley is considered to have Ethiopia as its center of diversity, and it is among the most prominent cereal crops cultivated across different agroecology in the country. However, the available germplasm in the country has not been studied much compared to the number of accessions under conservation and the expected diversity of crops in the country. This study was therefore conducted to estimate the phenotypic variability and association of morphoagronomic traits among 49 barley accessions. The experiment was conducted in 2021 using a 7 × 7 simple lattice design. The results of the analysis of variance indicated significant differences among the accessions for all traits. Moreover, with a mean of 4.02 t·ha^−1^, the variation in accessions for grain yield ranged from 2.18 to 6.89 t·ha^−1^. The phenotypic and genotypic coefficients of variation varied in the range between 7.25% (days to maturity) and 35.18% (weight of kernels per spike) and 6.61% (peduncle length) and 32.25% (weight of kernels per spike), respectively. Broad-sense heritability and genetic advance as a percentage of mean varied from 43.18 (number of fertile tillers) to 92.5% (days to heading) and 11.87% (peduncle length) to 60.99% (weight of kernel per spike), respectively. Grain yield had positive phenotypic and genotypic correlations with the majority of traits. Moreover, the number of spikelets per spike followed by the number of fertile tillers, thousand kernel weight, and number of kernels per spike had strong positive associations with grain yield and they had a direct, positive genotypic effect on grain yield. Consequently, while selecting accessions for high grain yields, these traits should be considered as well. The Euclidian distances of accessions estimated from quantitative traits ranged from 1.07 to 9.24, and the accessions were clustered into six distinct clusters. Clusters V (32.65%), II (26.5%), VI (24.49%), and IV (12.25%) consisted of the largest proportion of accessions, whereas Clusters I and III consisted of one accession each. From principal components' analysis, 79.65% of the variance was explained by three main components with eigenvalues greater than one. Thus, the current findings suggest that there is wide genetic variation among accessions which may be used for crop improvement and the information generated could also be utilized for genetic conservation.

## 1. Introduction

Barley (*Hordeum vulgare* L.) is one of the earliest domesticated cereal crops and originated around 10,000 years ago from its wild ancestor, *Hordeum spontaneum* (C. Koch) Thell, in the Fertile Crescent area of the Middle East [1, 2]. In terms of production and cultivated area, barley ranks fourth globally among cereal crops following maize, rice, and wheat [3]. It is cultivated worldwide in various agroecological zones and is mostly used for feed, brewing malts, and food purposes [4, 5]. European Union, Russia, and Australia are the leading barley-producing countries in the world with a whole production volume of 51.5, 21.5, and 13.7 million metric tons, respectively [6]. Ethiopia contributes approximately 34.5% of the total production in Africa, which makes it the second-largest producer on the continent [3].

Barley is cultivated in Ethiopia during different growing seasons at altitudes between 1800 and 3400 m.a.s.l. and it is currently the country's fifth most important cereal crop [7]. It is grown in most parts of the country; however, Oromia and Amhara Regional states, particularly; Arsi, Bale, Gojjam, Gonder, Shewa, Wellega, and Wello are the major barley-producing areas, accounting for more than 90% of the country's barley production [8]. It is cultivated on 799,127.84 ha of land and it has a total production of 2.04 million metric tons with remaining low productivity in the country as compared to the average yield achieved globally [8].

Barley is the top significant grain crop in Ethiopia and is used as a component of various health foods, as a source of beverages, as well as animal fodder and straw, which are used for construction by several smallholder farmers in the highlands [9, 10]. It is thought of as a poor man's crop used for the preparation of household food types such as “dabo,” “kolo,” “genfo,” “kinche,” and “beso” and local beverages such as “tela,” “borde,” and other [11]. Barley landraces cover nearly 90% of barley-growing areas in Ethiopia [12], and it has a vital role in food security in the country. However, the mean yield is low (2.11 t·ha^−1^) [13] compared with the average global productivity (3.1) t·ha^−1^ [3]. Therefore, for food security, increasing the productivity and total production of barley is a critical measure to be taken in Ethiopia. Increasing the production of barley by increasing the area cultivated for barley production seems difficult because the highlands and mid-highlands of Ethiopia, where barley is largely produced, are densely populated [14] and arable land is almost fully utilized. Thus, a better option to increase barley production is to develop varieties with high yields and other desirable traits. The development of high-yielding varieties requires the presence of genetic variation or creating variation within the crop and knowing the extent and nature of existing genetic variation.

Studying the existing genetic variability between germplasm accessions is the most important step in providing predictive estimates of genetic variation for efficient germplasm management and utilization and selection of parental accessions for breeding and development of an efficient breeding program. Since a high genetic variability of the germplasm increases the opportunity for breeders to select accessions specifically for the trait of interest [15], the global genetic variability of barley can be detected through diversity studies involving numerous landraces and cultivars [16, 17]. Studies on barley landraces from India [18], Pakistan [19], Brazil [20], and Jordan [21] indicate substantial genetic variability, which is indispensable for yield improvement. Previous studies have shown significant levels of genetic variability in Ethiopian barley accessions from different geographic origins [22–26]. However, a gap still exists in the preliminary characterization and evaluation of germplasm accessions to explore the extent of association among morphoagronomic traits, resulting in a lack of adequate information and their utilization in barley breeding. Exploring the extent of associations among traits requires the use of techniques such as path coefficient analysis, which divides the correlation coefficients into direct and indirect effects [27]. This simplifies the selection of accession for complex traits such as yield.

Therefore, information concerning phenotypic variability and the relationship between morphoagronomic traits among barley accessions is needed. Currently, more than 17,000 accessions of barley from various parts of the country are under conservation by the Ethiopian Biodiversity Institute (https://ebi.gov.et/biodiversity/conservation/genetic-material-holdings), among which genetic variability assessments and association were conducted in a very small proportion. Thus, the study was conducted to assess the genetic variability and association of morphoagronomic traits among barley accessions.

## 2. Materials and Methods

### 2.1. Description of Study Site

The research was conducted at the Holetta Agriculture Research Center. The research station was located at 09° 03′ N latitude and 38° 30′E longitude in the west direction from Addis Ababa at an altitude of 2400 m.a.s.l. The site's average yearly minimum and maximum temperatures were 6.7°C and 21.7°C, respectively. With a bimodal distribution, the area obtains an average yearly rainfall of 1141.1 mm, 70% of which falls during the main rainy season and 30% during the small rainy season. The soil at the experimental site is clay, which can be grouped as Nitisol, with a pH of 6.2 (https://www.eiar.gov.et/holetta).

### 2.2. Experimental Materials and Design

**Figure 1:**
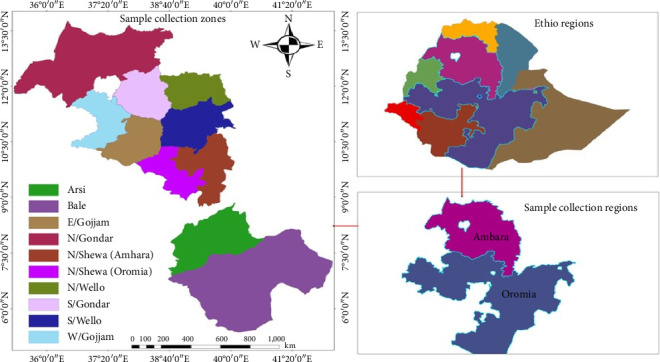
Map showing the administrative regions and zones of Ethiopia where the barley accessions used in this study were collected. E: east, N: north, S: south, and W: west.

In total, 49 barley accessions (including both two-row, irregular, and six-row) were evaluated. Accessions were collected from the parts of the Amhara and Oromia Regions by the Ethiopian Biodiversity Institute (Figure 1; Table S1). The accessions were planted in July 2021 using simple lattice designs. A seed rate of 100 kg/ha was used and sown by hand drilling. Each accession was sown in four rows of 2 m long, and each was separated by 0.2 m between rows and 0.5 m between plots. The spacing between the blocks and replicates was 1 m and 2 m, respectively.

### 2.3. Data Collection

Data were collected for morphoagronomic traits on both plant and plot bases, using the barley descriptor of International Plant Genetic Resources [28]. Fifteen randomly selected plants from the middle rows of the experimental plots were used to collect data on a plant basis, whereas the whole experimental plots were used to collect data on a plot basis. Data were collected from sample plants for the number of fertile tillers (the number of fertile tillers exclusively of the main plant was counted and estimated from 1 m length of the central row), plant height (measured from the ground surface to the spike's tip in centimeter, excluding awns at physiological maturity), peduncle length (measured from the uppermost node to the base of spikes), spike length (measured in centimeters from the base to the tip of the spike, excluding awns), number of spikelets per spike (scored by counting the number of spikelets from individual spikes), number of kernels per spike (seeds from each spike were threshed and counted), and weight of kernels per spike (weight of seeds harvested from randomly tagged individual spikes was measured in grams). Data were collected on a plot basis for days to heading (recorded by counting the number of days from sowing to the heading of 50% of plants in each plot), days to maturity (scored by counting the number of days from sowing to 75% of plants reaching the physiological maturity), thousand kernel weight (1000 randomly selected kernels from each plot were weighed in grams and transformed to standard grain moisture content (12.5%)), grain yield (seeds obtained from all plants in each plot were weighed by analytical balance and adjusted to 12.5% moisture content).

### 2.4. Statistical Analysis

#### 2.4.1. Analysis of Variance (ANOVA)

To estimate the variation among the accessions, the quantitative data were subjected to ANOVA in R software [29] using the PBIB.test function and the model variance component from the agricolae package [30]. The comparison of the mean performance of accessions was performed following the significance of mean squares using Tukey's test at a 5% probability level. The traits that showed significant mean squares in the general ANOVA were used for further analyses.

#### 2.4.2. Estimate of Variability Components

The genotypic and phenotypic variances and coefficients of variation of each quantitative trait were computed [31] considering mean square expectations from a simple lattice ANOVA as follows:

(1) $$\begin{array}{c} \sigma^{2}g=\left[\frac{K+1}{\text{kr}}\right]\left[\text{Msg}-\text{Mse}\right],\sigma^{2}p=\sigma^{2}g+\sigma^{2}e, \end{array}$$

where *σ*^2^*g* is the genotypic variance, Msg is the genotype mean square, Mse is the error mean square, *r* is the number of replications, *k*  is the number of plots within the block, *σ*^2^*p* is the phenotypic variance, and *σ*^2^*e*  is the environmental variance.

(2) $$\begin{array}{c} \text{GCV}\left(\%\right)=\left[\frac{\sqrt{\sigma^{2}g}}{\;\bar{X}}\right]\times 100,\\ \text{PCV}\left(\%\right)=\left[\frac{\sqrt{\sigma^{2}p}}{\;\bar{X}}\right]\times 100, \end{array}$$

where PCV is the phenotypic coefficient of variation, GCV is the genotypic coefficient of variation, and $\bar{X}$ is the population means of the character being evaluated and the range was classified into low, medium, and high values as indicated by the authors in [32].

Heritability in a broad sense was calculated by using the following formula [33]:

(3) $$\begin{array}{c} H^{2}=\left(\frac{\sigma^{2}g}{\sigma^{2}p}\right)\times 100, \end{array}$$

where H^2^ is the heritability in a broad sense, *σ*^2^p is the phenotypic variance, and *σ*^2^g is the genotypic variance, and it was categorized as suggested by the authors in [34].

Genetic advance (GA) in the absolute unit and genetic advance as the percentage of the mean (GAM) were calculated by using the following formula [35]:

(4) $$\begin{array}{c} \text{GA}=K\times \sigma p\times H^{2},\\ \text{GAM}=\left(\frac{\text{GA}}{\bar{X}}\right)\times 100, \end{array}$$

where GA is the GA, *σ*p is the phenotypic standard deviation on a mean basis, H^2^ is the heritability in the broad sense, and K is the standardized selection differential at 5% selection intensity (*K* is the  2.063). GAM is the GAM, $\bar{X}$ is the populations' mean character being evaluated. According to Johnson [35], the values of broad-sense heritability and GA as a percentage of the mean were categorized as low, moderate, and high.

#### 2.4.3. Estimate of Correlation and Path Coefficient Analysis

Phenotypic and genotypic correlation coefficients were estimated from the corresponding phenotypic and genotypic variances and covariances of the characters using the formula adopted by the authors in [36]. Path coefficient analysis was used to partition the correlation coefficients to the direct and indirect effects of the characters on yield, as per the method suggested by the authors in [37].

#### 2.4.4. Principal Component Analysis

Principal component analysis was conducted after the agromorphological traits with a significant mean square in the general ANOVA were homogenized to mean zero and variance of unity. The principal component based on the correlation matrix was calculated using the packages FactoMinor [38], factoextra [39], ggplot2 [40], and ggrepel [41] of R software Version 4.3.1 [29] and the values were taken as interns of eigen values and eigen vectors.

#### 2.4.5. Genetic Distance and Clustering

The genetic distance of 49 barley accessions was calculated using the Euclidean distance (ED) estimated from standardized quantitative traits, as per [42]. Clustering was performed based on the unweighted pair-group method with arithmetic means (UPGMA) using a distance matrix from agromorphological traits in XLSTAT software. The cluster analysis results are presented in the form of a dendrogram. Similarly, mean ED was computed for each accession by averaging a particular accession with the other 48 accessions to determine which accessions were closest or distant to others.

## 3. Results and Discussion

### 3.1. ANOVA

ANOVA showed highly significant (*p* < 0.01) differences among barley accessions for days to heading, days to maturity, plant height, peduncle length, spike length, number of spikelets per spike, number of kernels per spike, weight of kernels per spike, thousand kernel weight, and grain yield. Also, the number of fertile tillers exhibited significant differences (*p* ≤ 0.05) (Table 1). The significant differences among accessions indicate the existence of potential genetic variation among accessions, which can be used as a source of genetic material for barley improvement purposes.

Previous studies have reported highly significant variation in barley accessions for the majority of traits. Addisu and Shumet [43]; Aklilu [44]; Dido et al. [45], and Geleta, Dagnachew, and Zerihun [27] reported significant variations in days to maturity, number of fertile tillers, plant height, peduncle length, spike length, number of spikelets per spike, number of kernels per spike, thousand kernel weight, and grain yield from 36 to 585 barley accessions. In contrast to the results of this study, nonsignificant differences among barley accessions have been reported for fertile tillers per plant [46], spike length [24, 46], and plant height [13].

### 3.2. Mean Performances of the Barley Accessions

The mean performance of the accessions for morphoagronomic traits is displayed in Table S2. The accessions showed variations for days to heading and maturity in the range between 60 and 85 and 98 and 129, respectively. Accessions 4426 and 4366, which are two-rowed, showed early days to heading (60 days) and maturity (98 days), respectively, which might be useful for breeding to develop varieties that escape water stress during grain-filling periods [47]. Likewise, Accession 243,599 showed notably late days to heading (85 days) and maturity (129 days). Comparatively, 32 (65.31%) and 39 (79.59%) of accessions showed intermediate days to heading (65.5–79 days) and maturity (104–123 days), as compared to accessions that showed early days to heading and maturity as well. Other authors have also reported the existence of noticeable variations among barley landraces for days to heading and days to maturity, ranging from 80.2 to 85.5 and 138 to 144.3 [48] and from 74.3 to 85 and 117 to 137 [26].

The mean values of accessions for plant height and peduncle length varied from 72.3 to 126.4 and 31.6 to 43.3, in the same order. The tallest accession was 4423 (126.4 cm), followed by 8526 (116.9 cm) and 243,599 (111.7 cm), while the shortest accession was 243,287 (72.3 cm), followed by 243,288 (78.6 cm) and 243,286 (79.2 cm). The accessions with the shortest and longest peduncle lengths were 243,231 and 9949, respectively. The accessions with the highest number of fertile tillers were 4427, 8556, and 243,215, whereas accessions with the smallest number of fertile tillers were 4423 and 243,600. Several authors have previously reported relatively closer ranges of variation in barley genotypes for plant height, peduncle length, and the number of fertile tillers [49–51].

Spike length ranged from 5.74 to 9.34 cm with a mean of 7.87 cm. The accessions with the shortest and longest spike lengths were 243,214 and 243,599, respectively. The mean spike length of the remaining 28 accessions was 7.97–9.34 cm longer than the overall mean spike length of the accessions (7.87 cm), while the mean spike length of the 19 accessions was 5.93–7.77 cm shorter than the overall mean spike length of the barley accessions. Previous studies have reported spike lengths ranging from 3 to 14.4 for barley genotypes collected from various countries [16] and 6.44 to 15.55 for barley genotypes collected from various agroecological zones of Turkey [52]. Similarly, Ebrahim, Shiferaw, and Hailu [26] reported spike lengths ranging from 5.33 to 9.38 with a mean spike length of 6.93 cm. The ranges of the mean values for the number of spikelets per spike, number of kernels per spike, and weight of the kernels per spike were 17.57–25.76, 21.6–56.43, and 0.73–2.7, respectively. Two-row and six-row barley are the two major subspecies of *Hordeum* distinguished by their spike morphology and the number of fertile spikelets at each node of the rachis. Six-row barley has three fertile spikelets, while in two-row barley, only the central spikelets are fertile [1, 53]. Accession 243,286, a two-rowed barley, had the lowest mean number of spikelets per spike, the number of kernels per spike, and the weight of kernels per spike. The highest means for the number of spikelets per spike, number of kernels per spike, and weight of kernels per spike were noted for six-rowed barley as 243,229, 243,215, and 243,289, respectively. Closer ranges and mean values have been reported for spikelets per spike by the authors in [13, 26]. Bekele, Yoseph, and Ayalew [11] observed that the average number of kernels per spike is 46.10. Hu, Barmeier, and Schmidhalter [54] reported that the weight of kernels per spike varies from 0.88 to 1.10.

The accessions had mean values for thousand kernel weights and grain yield ranged from 27.79 to 48.70 g and 2.28 to 6.06 t·ha^−1^, respectively. The lowest thousand kernel weight and grain yield were obtained from 243,231, and the highest thousand kernel weight and grain yield were recorded from 243,229. Similarly, other authors [13, 26, 55] reported a large range of variation in the grain yield of barley genotypes. Assefa et al. [56] recorded up to 45.13 g of thousand kernel weight from eight barley genotypes.

### 3.3. Estimates of Variability Components

The PCV and GCV were computed to estimate the level of genetic variation for environmental and genetic factors. The PCV ranged from 7.25% to 35.18%, whereas the GCV ranged from 6.61% to 32.25% (Table 2). High PCV and GCV were observed for the weight of kernels per spike, grain yield, and number of kernels per spike. In agreement with the present study [43, 48], high GCV and PCV values for grain yield and number of kernels per spike were estimated. Moderate PCV coupled with moderate GCV was observed for plant height, number of fertile tillers, spike length, and thousand kernel weight. However, low PCV and GCV values were estimated for days to heading, days to maturity, peduncle length, and the number of spikelets per spike (Table 2).

The difference between the PCV and GCV values was low for the majority of traits, even though our experiment was limited to a specific year and location. This implies that the majority of traits were less influenced by environmental factors [17]. Dinsa, Mekbib, and Letta [57] and Hailu et al. [58] observed high values of PCV and GCV for grain yield of barley genotypes, moderate values for thousand kernel weight, and low values for days to heading and days to maturity. Yadav et al. [59] reported high GCV and PCV for grain yield and grain weight per spike, intermediate GCV and PCV for spike length and plant height, and low GCV and PCV for days to heading and days to maturity. Dido et al. [25] observed high GCV and PCV values for plant height and the number of fertile tillers, intermediate PCV for days to heading and days to maturity, and low GCV for the number of kernels per spike for all accessions studied, which contradicts the current result.

The values of broad-sense heritability were estimated in the range between 43.18% and 92.5%. High heritability values were obtained for all of the quantitative traits studied except for the number of fertile tillers (43.18%), which had moderate heritability (Table 2). GA as a percentage of the mean ranged from 11.87% to 60.99% for peduncle length and weight of kernel spike per spike, respectively. High GAM was found for the weight of kernels per spike, grain yield, number of kernels per spike, thousand kernel weights, spike length, and plant height. Moderate values of GAM were recorded for days to heading, number of fertile tillers per plant, number of spikelets per spike, days to maturity, and peduncle length (Table 2).

These results are in congruence with earlier reports [25, 43, 48, 59, 60], which observed moderate to high values of broad-sense heritability and GAM for characters such as days to heading, days to maturity, plant height, peduncle length, spike length, number of kernels per spike, number of spikelets per spike, thousand kernel weight, and grain yield. However, low heritability values have been reported for grain yield [61, 62], spike length [63], and peduncle length [57], which contradicts the present results.

Traits such as weight of kernels per spike, grain yield, number of kernels per spike, thousand kernel weight, spike length, and plant height exhibited high or moderately high levels of variance along with high heritability and GA (Table 2). This indicates that the traits are less influenced by the environment in their expression, heritability is most probably caused by additive gene effects, and phenotypic performance–based selection may be more successful. In addition, days to maturity have a low GCV and GA, which indicates the presence of environmental effects on these traits compared to other traits. These are influenced by nonadditive gene interactions and can be improved by breeding methods other than selection. Similar findings have been reported in various studies [25, 43, 48, 60].

### 3.4. Phenotypic and Genotypic Correlation Coefficient

Grain yield revealed a positive and highly significant association with the number of fertile tillers, number of spikelets per spike, number of kernels per spike, and thousand kernel weight at the genotypic and phenotypic levels (Table 3). It showed a positive and significant association with days to heading and plant height at both genotypic and phenotypic levels. Highly significant positive associations were reported for days to maturity and kernel weight per spike with grain yield at the phenotypic level, and significant positive associations were observed for those traits at the genotypic level, which is in agreement with the studies of authors in [27, 44, 48, 64], who reported that grain yield was positively and significantly associated with days to heading, days to maturity, plant height, productive tillers per plant, grain weight per spike, number of spikelets per spike, number of kernels per spike, and thousand seed weight at the genotypic and phenotypic levels.

Kumar et al. [65] observed that grain yield per plant had a highly significant and positive genotypic association with thousand grain weight, plant height, fertile tiller per plant, and weight of grain per main spike. Dido et al. [25] and Güngör et al. [66] also found positive and significant correlations between grain yield and days to heading, days to maturity, fertile tillers per plant, and number of seeds per spike. The estimates of genotypic and phenotypic associations revealed the presence of significant positive correlations among phenological traits (days to heading and days to maturity) and yield-related traits, such as the number of spikelets per spike, number of kernels per spike, and weight of kernel per spike. In addition, plant height and number of fertile tillers per plant were positively and significantly correlated with grain yield and yield components, such as the number of spikelets per spike, number of kernels per spike, weight of kernel per spike, and thousand kernel weight (Table 3). This indicates that plant height and number of fertile tillers per plant are traits directly related to the yield potential of the plant. Consistent with our results [25, 44], positive and significant genotypic and phenotypic correlations were observed between phenological traits (days to heading and days to maturity) and the number of seeds per spike.

Geleta, Dagnachew, and Zerihun [27] found highly significant and positive genotypic and phenotypic associations between plant height and the number of fertile tillers with kernel weight per spike, spikelets number per spike, and thousand kernel weight. Mekasa and Mohammed [64] observed a significant positive correlation between both plant height and the number of fertile tillers per plant with the number of kernels per spike and days to maturity at the genotypic and phenotypic levels. However, a significant negative genotypic and phenotypic correlation between plant height and days to maturity was also reported by the author, which contradicts the current result. The significant and positive association between the tillers number per plant with the grain number per spike and thousand kernel weight was reported by the authors in [67].

Spikelet length showed a negative and significant association at the genotypic and phenotypic levels with the number of kernels per spike and the weight of kernels per spike, whereas it showed a positive and significant correlation with peduncle length. In agreement with this finding [68], a negative and significant correlation was found between spikelet length and the number of kernels per spike at the phenotypic level. Yield-related traits, such as the number of spikelets per spike, number of kernels per spike, weight of kernels per spike, and thousand kernel weight had a positive and strong correlation at both genotypic and phenotypic levels, except for the genotypic correlation between thousand kernel weight and weight of kernels per spike. In the same manner, positive and highly significant phenotypic correlation among the number of spikelets per spike, number of kernels per spike, and weight of kernels per spike has been reported by the authors in [69]. Earlier studies also observed strong positive genotypic and phenotypic correlations between thousand kernel weight and number of spikelets per spike [27] and thousand kernel weight with the number of kernels per spike [25], which consolidates the current results.

### 3.5. Path Coefficient Analysis

#### 3.5.1. Genotypic Path Coefficient Analysis

Path coefficient analysis examines the direct and indirect effects of independent variables on dependent variables, which correlation coefficient analysis may not be able to provide [37]. Grain yield was used as the dependent variable and the other traits were used as independent variables in the path coefficient analysis. In this study, only the traits that showed significant correlations with grain yield were included in the path analysis [37]. The direct effects of path coefficient analysis indicated that the grain yield of barley accessions was positively affected by phenology, growth, and yield-related traits, such as days to heading, days to maturity, number of fertile tillers, number of spikelets per spike, number of kernels per spike, and thousand kernel weight (Table 4).

According to the authors in [70], the direct and indirect effects were categorized as very high (> 1.00), high (0.30–1.00), moderate (0.20–0.29), low (0.10–0.19), and negligible (0.00–0.09). In the present study, genotypic path coefficient analysis showed that the number of kernels per spike (0.324) followed by thousand kernel weight (0.308) exerted a high and positive direct effect on grain yield, which is in agreement with the authors of [69], who found a highly positive genotypic direct effect of the number of kernels per spike and thousand kernel weight on grain yield. The number of fertile tillers (0.271) and spikelets per spike (0.225) had moderate and positive direct effects on grain yield. The current results are also in line with those of the genotypic path analysis reported by the authors in [25], who found that the number of fertile tillers per plant and the number of spikelets per spike had a positive direct effect on grain yield.

Days to maturity (0.136) and days to heading (0.096) exerted low and negligible positive direct effects, respectively, on grain yield (Table 4). Even if their direct effect was small, the cumulative effects of other traits through indirect effects led to a positive and significant correlation with grain yield (Table 3). As a result, while selection is undertaken for these traits, their indirect effects on other traits should also be considered. On the other hand, plant height and kernel weight per spike exerted a negative direct effect on grain yield. However, they had a positive and significant association with grain yield because of the indirect effects exerted through other traits. Aklilu, Dejene, and Worede [44] found a negative and negligible direct effect of plant height and grain yield at the genotypic level, which agrees with the current results. In contrast, the authors in [27] reported a negative direct effect of days to heading and days to maturity on grain yield at the genotypic level. The residual effect from genotypic path analysis was 0.077 (Table 4), which revealed that 92.23% of the variation in grain yield was explained by the traits studied in this experiment. The remaining 7.77% are explained by other traits which were not included in this study.

#### 3.5.2. Phenotypic Path Coefficient Analysis

Thousand kernel weight (0.388) and number of kernels per spike (0.355), which had a positive and highly significant correlation with grain yield, showed a high direct effect on grain yield (Table 5). These indicate that both traits had a true association with grain yield. Therefore, when working on selection aimed at improving grain yield, important consideration should be given to these traits. These results are in accordance with those of [71], which reported the highest direct effect of thousand grain weight and number of grains per spike on grain yield under irrigated conditions. In the present study, the number of spikelets per spike (0.233) had a moderate positive direct effect on grain yield. Besides this, positive and small direct effects on grain yield were exerted by days to heading, days to maturity, and the number of fertile tillers per plant. The association between the weight of kernels per spike and plant height was positive and significant; however, they had a negative direct effect on grain yield. This indicates that the negative direct effect is influenced by the counterbalance indirect effect of the weight of kernels per spike via the number of kernels per spike (0.268) and plant height through days to maturity (0.154). Hailu et al. [58] and Tahar et al. [72] reported a direct negative phenotypic effect of plant height on grain yield which supports the present results. Geleta, Dagnachew, and Zerihun [27] also reported a positive direct effect of days to heading and days to maturity and a negative direct effect of grain weight per spike on grain yield, which agrees with the present result. The residual effect from phenotypic path analysis was 0.1623 (Table 5), which revealed that 83.77% of the variability in grain yield was explained by the traits studied in this experiment. The remaining 16.23% was covered by other traits that were not included in the study.

### 3.6. Genetic Distance and Cluster Analyses

#### 3.6.1. Genetic Distances Among Barley Accessions

The genetic distances for all possible pairs of 49 barley accessions are presented in Table S3. The genetic distances of accessions varied from 1.07 to 9.24, with the mean, standard deviation, and coefficient of variation of 4.44%, 1.50%, and 33.8%, respectively (Table 6). The ED between the barley accessions 243,215 and 243,286 (9.24) was the highest, followed by 243,286 and 243,599 (9.07) and 243,215 and 243,287 (8.93). While the lowest genetic distance was found between 243,215 and 243,229 (1.07), followed by 8525 and 232,222 (1.33) and 232,222 and 243,213 (1.39) (Table S3). To obtain information regarding the most distant and closest accessions, the average genetic distance of every barley accession to the other 48 accessions was computed (Table 6). Based on the mean ED, 243,231 (5.69), 4427 (5.64), and 243,215 (5.54) were the most distant accessions, whereas 232,222 (3.40), 235,066 (3.52), and 243,213 (3.59) were found to be the closest to other accessions. In general, almost 50% of accessions had a mean genetic distance of > 4.44 (overall mean distances of accessions). These findings indicate the presence of genetically distant accessions which can be used to combine the most desirable traits in progeny through hybridization [23]. Similarly, Monteiro et al. [73] estimated the genetic distances of 435 Brazilian barley accessions with the genetic distances ranging from 0.025 to 0.572, with a mean of 0.256.

#### 3.6.2. Clustering of Accessions

**Figure 2:**
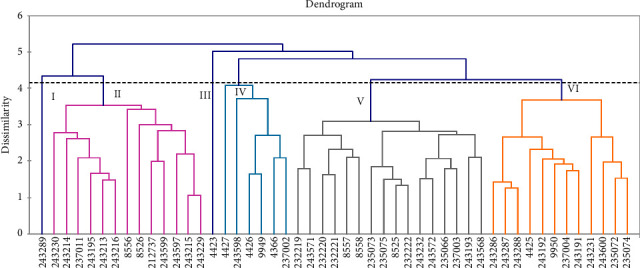
Dendrogram depicting dissimilarity of 49 barley accessions estimated for 11 quantitative traits.

Cluster analysis based on the UPGMA clustering method from the ED matrix divided 49 barley accessions into six main clusters. Cluster V was the largest cluster consisting of 16 accessions (32.65%). Clusters II and VI consisted of 13 (26.5%) and 12 (24.49%) accessions, respectively, and Clusters I and III were constructed with one accession each (Figure 2; Table 7). Most of the accessions (83.67%) were classified into three clusters. Clusters II and VI comprised accessions from Arsi, Bale, North Shewa, Gonder, and Gojjam, whereas Cluster VI consisted of accessions from Wollo, Arsi, North Shewa, and Bale (Table 7). Cluster analysis clustered accessions with higher phenotypic similarity. However, the clusters did not necessarily include all accessions collected from the same or neighboring regions. These results are consistent with those of previous reports [22, 24].

Bedasa, Berhane, and Tadesse [24] examined 102 Ethiopian barley accessions using five standard checks and the genotypes were grouped into five clusters, whereas the authors in [72] studied 36 barley genotypes and classified them into seven clusters. Forty-eight Ethiopian barley landraces were evaluated by the authors in [74] and they were categorized into six clusters. In addition, the authors in [75] evaluated 36 barley landraces collected from southern Ethiopia and grouped them into four clusters. Derbew [13] also grouped 22 genotypes into five groups.

#### 3.6.3. Cluster Mean Analysis

The mean values of the six clusters for 11 morphoagronomic traits are displayed in Table 8. The mean values were higher in Cluster I for days to maturity, spike length, number of spikelets per spike, number of kernels per spike, weight of kernels per spike, thousand kernel weight, and grain yield. Cluster II had a greater mean value than the overall mean for days to heading, days to maturity, plant height, number of fertile tillers, number of spikelets per spike, number of kernels per spike, weight of kernels per spike, thousand kernel weight, and grain yield. In Cluster III, days to maturity, plant height, peduncle length, spike length, and thousand kernel weight had mean values higher than the overall mean. Cluster IV differentiated by having higher mean values for all traits, including grain yield, than the overall mean values of accessions, except for days to heading, days to maturity, number of kernels per spike, and weight of kernels per spike. The low values of days to heading and days to maturity in this cluster indicated that the accessions matured early. As a result, continuing to test members of this cluster to produce early maturing varieties may be a possible option in areas where terminal drought is a constraint on barley production.

Cluster V had a higher mean number for days to heading, plant height, peduncle length, number of kernels per spike, and weight of kernels per spike. Cluster VI had a mean spike length greater than the overall mean. However, for grain yield and other traits, this cluster showed lower mean values than the overall mean values of accessions. The cluster mean analysis showed the possibility of selection of accessions for varied desirable traits from Clusters I, II, and IV. Several authors also found that some clusters formed by barley landraces collected from Ethiopia showed greater mean values for desirable traits [13, 24, 45, 74, 76].

#### 3.6.4. Principal Component Analysis

The results of principal component analyses for 11 quantitative traits are shown in Table 9. As Holland [77] suggested, standard criteria allowed excluded components whose eigenvalues are less than 1 when a correlation matrix is used. In this study, the analysis of principal components showed three principal components with eigenvalues larger than one, which accounted for approximately 79.65% of the total variation for 11 traits. Similarly, Derbew [13]; Dido et al. [45]; and Ebrahim, Shiferaw, and Hailu [26] reported that the first three principal components contributed to about 77.6%, 51.75% and 84.22% of the total variation in barley landraces, respectively. The first principal component individually accounted for 47.79% of the total variability because of relatively high loading from grain yield, number of kernels per spike, number of spikelets per spike, weight of kernel per spike, number of fertile tillers, thousand kernel weight, and days to maturity. The second principal component contributed 20.54% of the total variation due to loading from spike length, thousand kernel weight, peduncle length, days to heading, and weight of kernels. The third principal component also indicated variation owing to high loading from peduncle length, plant height, and number of fertile tillers. The traits having a greater contribution to the total variation of each principal component implied that these traits induced barley accessions to separate into different clusters. Fantahun et al. [78] reported that days to maturity and number of kernels per spike were the highest contributors to the variation observed between 320 barley genotypes evaluated.

**Figure 3:**
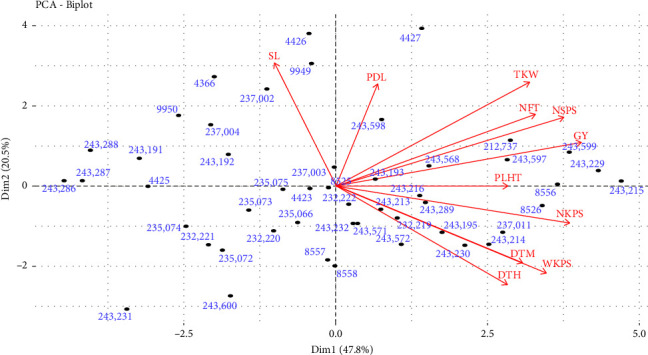
Biplot accession trait indicated the contribution of accessions and traits to PCA1 and PCA2 for 11 traits. DTH: days to heading, DTM: days to maturity, GY: grain yield, NFT: no. of fertile tiller, NKPS: no. of kernels per spike, NSPS: no. of spikelet per spike, PDL: peduncle length, PLHT: plant height, SL: spikelet length, TKW: thousand kernel weight, and WKPS: weight of kernels per spike.

A biplot was constructed based on the first and the second PCA (Figure 3). The accessions and traits are presented on a biplot to show their relationships. The first two PCA biplots explained 68.33% of the overall variation between genotypes, indicating that thousand kernel weight, number of spikelets per spike, grain yield, number of kernels per spike, seed yield weight of kernels per spike, and spike length were the most discriminating traits. The accessions in the right top quadrant had the highest thousand kernel weights, number of spikelets per spike, number of fertile tillers, and grain yield, which is comparable with the previous study [78, 79]. The accessions located in the bottom right quadrant were identified by having a relatively late heading and maturity date, as well as a high number of kernels per spike and a high weight of kernels per spike. The accessions scattered near to the origin had a common genetic characteristic, conversely, the accessions located far from the origin are thought to be unrelated accessions [74]. Thus, these divergent accessions can be used in barley breeding programs as potential sources for successful hybridization to develop heterotic groups.

## 4. Conclusion

Barley is a prominent cereal crop cultivated in various agroecological conditions in Ethiopia. It has a significant role in the country's food security. However, the mean yield is low compared with other barley countries, which proposes that further research is needed to increase the productivity of barley crops through high-yielding varieties. Ethiopia is the center of diversity and is rich in large collections of barley accessions. Therefore, understanding the nature and magnitude of the existing variation makes it possible to utilize genetic variability in breeding programs and germplasm conservation. Generally, the results of the research showed the existence of broad variations among barley accessions for grain yield and yield-related traits; the majority of the traits exhibited high heritability, GA, GCV, and PCV, and most of the traits had positive and significant associations with grain yield. Almost 50% of accessions had a mean genetic distance greater than the overall mean distances of accessions. These major findings indicate that Ethiopian barley accessions have a high probability of producing new varieties through selection and/or hybridization. However, the current study was performed for one season at a single location for morphoagronomic traits. Thus, it is suggested that the evaluation of accessions in different locations and years for morphoagronomic traits combined with molecular markers will be needed to make tangible conclusions.

## Figures and Tables

**Table 1:** Mean square from analysis of variance for 11 quantitative traits.

Source of variation	df	DTH	DTM	PLHT	PDL	NFT	SL	NSPS	NKPS	WKPS	TKW	GY
Replication	1	32⁣^∗∗^	34.33⁣^∗^	530.17⁣^∗∗∗^	0.83ns	0.18ns	1.56⁣^∗^	11.55⁣^∗∗^	48.84ns	0.93⁣^∗∗∗^	0.09Ns	0.35Ns
Accession	48	93.6⁣^∗∗∗^	114.68⁣^∗∗∗^	205.26⁣^∗∗∗^	12.3⁣^∗∗∗^	0.48⁣^∗∗^	1.73⁣^∗∗∗^	7.68⁣^∗∗∗^	165.93⁣^∗∗∗^	0.58⁣^∗∗∗^	59.91⁣^∗∗∗^	2.03⁣^∗∗∗^
Block	12	10.56⁣^∗^	69.90⁣^∗∗∗^	98.83⁣^∗∗∗^	2.47ns	0.19ns	0.78⁣^∗∗^	1.48Ns	28.64ns	0.09Ns	5.83Ns	0.25Ns
Residual	36	4.15	5.5	13.95	1.9	0.21	0.25	1.2	19.72	0.06	4.93	0.16
CV (%)		2.8	2.1	3.8	3.7	14.6	6.4	5	10.8	14.1	5.9	10

*Note:*⁣^∗∗∗^, ⁣^∗∗^ and significant at *p* < 0.01, *p* < 0.05, and *p* < 0.1 respectively.

Abbreviations: CV (%), coefficient of variation in percent; df, degrees of freedom; DTH, days to heading; DTM, days to maturity; GY, grain yield; NFT, number of fertile tillers; NKPS, number of kernels per spike; ns, non significant; NSPS, number of spikelets per spike; PDL, peduncle length; PLHT, plant height; SL, spike length; TKW, thousand kernel weight; WKPS, weight of kernels per spike.

**Table 2:** Analyses of variability components for eleven morphoagronomic traits of barley accessions.

Traits	Range	Mean ± SEM	*σ* ^2^g	*σ* ^2^p	GCV (%)	PCV (%)	H^2^b (%)	GA	GAM (%)
DTH	60.0–87.0	71.7 ± 0.71	51.12	55.27	9.97	10.36	92.50	14.19	19.78
DTM	97.0–130	113.7 ± 0.83	62.39	67.89	6.95	7.25	91.90	15.62	13.74
PLHT	69.4–128	97.5 ± 1.12	109.3	123.3	10.7	11.39	88.68	20.31	20.84
PDL	29.1–43.9	36.9 ± 0.28	5.94	7.840	6.61	7.59	75.75	4.38	11.87
NFT	2.0–4.3	3.11 ± 0.06	0.16	0.360	12.7	19.32	43.18	0.54	17.21
SL	5.7–10.1	7.87 ± 0.10	0.85	1.100	11.7	13.31	76.81	1.66	21.09
NSPS	16.1–27.3	21.8 ± 0.21	3.70	4.900	8.84	10.17	75.53	3.45	15.85
NKPS	18.9–58.9	41.2 ± 0.97	83.6	103.3	22.2	24.65	80.90	16.96	41.83
WKPS	0.6–3.2	1.70 ± 0.06	0.30	0.360	32.3	35.18	84.05	1.04	60.99
TKW	25.7–51	37.4 ± 0.57	31.4	36.35	14.9	16.13	86.45	10.75	28.76
GY	2.2–6.9	4.02 ± 0.11	1.07	1.230	25.7	27.60	86.95	1.99	49.51

*Note:σ* ^2^g, genotypic variance; *σ*^2^p, phenotypic variance; H^2^b, broad-sense heritability.

Abbreviations: DTH, days to heading; DTM, days to maturity; GA, genetic advance; GAM, genetic advance as a percentage of mean; GCV, genotypic coefficient of variation; GY, grain yield; NFT, number of fertile tillers; NKPS, number of kernels per spike; NSPS, number of spikelets per spike; PCV, phenotypic coefficient of variation; PDL, peduncle length; PLHT, plant height; SL, spike length; TKW, thousand kernel weight; WKPS, weight of kernels per spike.

**Table 3:** Estimates of the phenotypic (below diagonal) and genotypic (above diagonal) correlation coefficients for 11 traits.

Traits	DTH	DTM	PLHT	PDL	NFT	SL	NSPS	NKPS	WKPS	TKW	GY
DTH	1	0.72⁣^∗∗^	0.45⁣^∗^	−0.07	0.20	−0.37	0.30⁣^∗^	0.56⁣^∗∗^	0.68⁣^∗∗^	0.09	0.39⁣^∗^
DTM	0.69⁣^∗∗^	1	0.43⁣^∗^	−0.23	0.27	−0.18	0.42⁣^∗^	0.54⁣^∗∗^	0.69⁣^∗∗^	0.26	0.51⁣^∗^
PLHT	0.38⁣^∗^	0.31⁣^∗^	1	0.46⁣^∗^	0.23	−0.09	0.45⁣^∗^	0.47⁣^∗^	0.56⁣^∗∗^	0.39⁣^∗^	0.40⁣^∗^
PDL	−0.07	−0.18	0.42⁣^∗∗^	1	0.17	0.30⁣^∗^	0.16	0.07	−0.06	0.34⁣^∗^	0.12
NFT	0.19	0.29⁣^∗^	0.16	0.16	1	−0.06	0.77⁣^∗∗^	0.56⁣^∗∗^	0.27	0.72⁣^∗∗^	0.85⁣^∗∗^
SL	−0.30⁣^∗^	−0.09	−0.13	0.30⁣^∗^	−0.02	1	0.13	−0.40⁣^∗^	−0.46⁣^∗^	0.19	−0.11
NSPS	0.29⁣^∗^	0.41⁣^∗∗^	0.42⁣^∗∗^	0.18	0.63⁣^∗∗^	0.12	1	0.62⁣^∗∗^	0.47⁣^∗^	0.79⁣^∗∗^	0.87⁣^∗∗^
NKPS	0.51⁣^∗∗^	0.48⁣^∗∗^	0.43⁣^∗∗^	0.06	0.44⁣^∗∗^	−0.31⁣^∗^	0.57⁣^∗∗^	1	0.81⁣^∗∗^	0.45⁣^∗^	0.73⁣^∗∗^
WKPS	0.64⁣^∗∗^	0.63⁣^∗∗^	0.52⁣^∗∗^	−0.05	0.25⁣^∗^	−0.35⁣^∗^	0.46⁣^∗∗^	0.76⁣^∗∗^	1	0.27	0.51⁣^∗^
TKW	0.06	0.24⁣^∗^	0.34⁣^∗^	0.31⁣^∗^	0.61⁣^∗∗^	0.16	0.70⁣^∗∗^	0.43⁣^∗^	0.26⁣^∗^	1	0.81⁣^∗∗^
GY	0.36⁣^∗^	0.47⁣^∗∗^	0.33⁣^∗^	0.11	0.72⁣^∗∗^	−0.09	0.78⁣^∗∗^	0.69⁣^∗∗^	0.46⁣^∗∗^	0.77⁣^∗∗^	1

*Note:*⁣^∗^ and ⁣^∗∗^, significant at *p* ≤ 0.05 and *p* ≤ 0.01, respectively.

Abbreviations: DTH, days to heading; DTM, days to maturity; GY, grain yield; NFT, number of fertile tillers; NKPS, number of kernels per spike; NSPS, number of spikelets per spike; PDL, peduncle length; PLHT, plant height; SL, spike length; TKW, thousand kernel weight; WKPS, weight of kernels per spike.

**Table 4:** Estimates of direct (bold diagonal) and indirect effect (off-diagonal) of 8 traits on grain yield at the genotypic level.

Traits	DTH	DTM	PLHT	NFT	NSPS	NKPS	WKPS	TKW	rg
DTH	**0.094**	0.098	−0.025	0.055	0.068	0.180	−0.101	0.028	0.397⁣^∗^
DTM	0.068	**0.136**	−0.024	0.073	0.095	0.174	−0.101	0.085	0.505⁣^∗^
PLHT	0.042	0.059	**−0.055**	0.062	0.102	0.153	−0.082	0.122	0.402⁣^∗^
NFT	0.019	0.037	−0.013	**0.271**	0.173	0.182	−0.041	0.221	0.849⁣^∗∗^
NSPS	0.028	0.058	−0.025	0.208	**0.225**	0.200	−0.069	0.246	0.870⁣^∗∗^
NKPS	0.052	0.073	−0.026	0.152	0.139	**0.324**	−0.120	0.138	0.733⁣^∗∗^
WKPS	0.064	0.094	−0.031	0.075	0.106	0.263	**−0.147**	0.084	0.507⁣^∗^
TKW	0.008	0.038	−0.022	0.195	0.179	0.145	−0.040	**0.308**	0.811⁣^∗∗^

*Note:* Residual = 0.077; ⁣^∗^ and ⁣^∗∗^ significant at *p* < 0.05 and *p* < 0.01, respectively.

Abbreviations: DTH, days to heading; DTM, days to maturity; NFT, number of fertile tillers; NKPS, number of kernels per spike; NSPS, number of spikelets per spike; PLHT, plant height; rg, genotypic correlation; TKW, thousand kernels weight; WKPS, weight of kernels per spike.

**Table 5:** Estimates of direct (bold diagonal) and indirect effect (off-diagonal) of 8 traits on grain yield at phenotypic level.

Traits	DTH	DTM	PLHT	NFT	NSPS	NKPS	WKPS	TKW	rp
DTH	**0.112**	0.071	−0.029	0.031	0.069	0.182	−0.101	0.025	0.360⁣^∗^
DTM	0.077	**0.103**	−0.023	0.048	0.095	0.171	−0.099	0.092	0.464⁣^∗∗^
PLHT	0.042	0.032	**−0.075**	0.026	0.097	0.154	−0.081	0.132	0.326⁣^∗^
NFT	0.021	0.030	−0.012	**0.167**	0.146	0.168	−0.039	0.235	0.716⁣^∗∗^
NSPS	0.033	0.042	−0.031	0.105	**0.233**	0.202	−0.072	0.273	0.784⁣^∗∗^
NKPS	0.056	0.050	−0.033	0.079	0.133	**0.355**	−0.119	0.167	0.689⁣^∗∗^
WKPS	0.072	0.065	−0.039	0.042	0.107	0.268	**−0.157**	0.100	0.457⁣^∗∗^
TKW	0.007	0.024	−0.026	0.101	0.164	0.152	−0.040	**0.388**	0.771⁣^∗∗^

*Note:* Residual = 0.1623; ⁣^∗^ and ⁣^∗∗^ significant at *p* < 0.05 and *p* < 0.01, respectively.

Abbreviations: DTH, days to heading; DTM, days to maturity; NFT, number of fertile tillers; NKPS, number of kernels per spike; NSPS, number of spikelet per spike; PLHT, plant height; rp, phenotypic correlation; TKW, thousand kernel weight; WKPS, weight of kernels per spike.

**Table 6:** Range, mean, standard deviation and coefficient of variation of genetic distances based on 11 quantitative traits.

Accessions	Min	Max	Mean	SD	CV (%)
4366	2.09	7.52	4.82	1.36	28.2
4423	4.04	7.15	5.25	0.88	16.78
4425	1.89	7.91	4.41	1.59	36.1
4426	1.65	7.80	5.04	1.22	24.24
4427	3.19	8.57	5.64	1.10	19.55
8525	1.33	5.54	3.65	1.07	29.26
8526	2.42	8.30	4.89	1.55	31.71
8556	2.68	8.26	5.30	1.58	29.79
8557	1.88	6.94	4.21	1.19	28.27
8558	1.99	7.22	4.35	1.26	28.9
9949	1.65	7.42	4.96	1.12	22.51
9950	1.87	7.54	4.55	1.43	31.53
212737	1.98	7.83	4.53	1.45	32.02
232219	1.78	6.95	4.29	1.19	27.6
232220	1.63	6.32	3.81	1.21	31.67
232221	1.63	7.27	4.22	1.47	34.82
232222	1.33	5.44	3.40	1.07	31.55
235066	1.68	5.82	3.52	1.12	31.83
235072	1.52	7.00	4.11	1.40	34.06
235073	1.68	6.52	3.94	1.26	32.07
235074	1.52	7.24	4.09	1.52	37.2
235075	1.47	5.86	3.60	1.11	30.76
237002	1.88	6.71	4.51	1.18	26.13
237003	1.80	5.41	3.58	0.82	22.92
237004	1.74	6.96	4.07	1.36	33.42
237011	1.76	7.53	4.31	1.54	35.84
243191	1.64	7.93	4.49	1.68	37.44
243192	2.01	6.54	3.93	1.17	29.80
243193	1.94	5.61	3.63	0.91	24.96
243195	1.55	6.59	4.03	1.33	32.95
243213	1.39	5.84	3.59	1.10	30.62
243214	2.06	7.30	4.64	1.43	30.90
243215	1.07	9.24	5.54	1.93	34.79
243216	1.47	6.27	3.84	1.14	29.66
243229	1.07	8.85	5.29	1.85	35.00
243230	2.07	6.95	4.62	1.34	28.98
243231	2.31	8.77	5.69	1.65	28.99
243232	1.51	5.30	3.57	1.06	29.84
243286	1.39	9.24	5.53	1.90	34.39
243287	1.26	8.93	5.47	1.85	33.72
243288	1.26	8.73	5.15	1.86	36.20
243289	3.42	6.89	5.00	0.91	18.28
243568	2.11	6.64	3.88	1.06	27.36
243571	1.78	6.19	4.07	1.15	28.13
243572	1.51	6.04	3.84	1.22	31.84
243597	1.98	7.47	4.60	1.41	30.69
243598	2.42	6.66	4.17	0.9	21.61
243599	1.98	9.07	5.54	1.61	28.99
243600	1.79	7.55	4.58	1.40	30.57
Overall	1.07	9.24	4.44	1.5	33.8

Abbreviations: CV, coefficient of variation; Max, maximum; Min, minimum; SD, standard deviation.

**Table 7:** The number of accessions grouped into six clusters, accessions, and collection area of barley accessions used in the study.

Clusters	No. and % of accessions	List of accessions	Area of collection
I	1 (2.04%)	243,289	Gonder

II	13 (26.5%)	8526, 8556, 212,737, 237,011, 243,195, 243,213, 243,214, 243,215, 243,216, 243,229, 243,230, 243,597, and 243,599	Arsi, Bale, North Shewa, Gojjam, and Gonder

III	1 (2.04%)	4423	Gojjam

IV	6 (12.25%)	4366, 4426, 4427, 9949, 237,002, and 243,598	Arsi, Gojjam, and Gonder

V	16 (32.65%)	8525, 8557, 8558, 232,219, 232,220 232,221, 232,222, 235,066, 235,073 235,075, 237,003, 243,193, 243,232, 243,568, 243,571, and 243,572	Wello, Arsi, North Shewa, and Bale

VI	12 (24.49%)	4425, 9950, 235,072, 235,074, 237,004, 243,191, 243,192, 243,231, 243,286, 243,287, 243,288, and 243,600	Gonder, Gojjam, Wello, Bale, Arsi, and North Shewa

**Table 8:** The mean value for six clusters based on eleven morphoagronomic traits of barley accessions.

Traits	Clusters						Overall mean
	C-I	C-II	C-III	C-IV	C-V	C-VI	
DTH	61.5	79.1	67	64.3	73.0	67.1	71.7
DTM	126.5	121.0	125.5	106.1	113.0	108.4	113.7
PLHT	92.3	103.2	126.4	97.9	100.3	85.3	97.5
PDL	32.6	36.3	37.9	40.7	37.6	34.9	36.9
NFT	3.10	3.70	2.3	3.4	3.0	2.70	3.10
SL	8.20	7.50	8.5	9.0	7.6	8.10	7.90
NSPS	23.5	23.8	20.9	23.0	21.1	19.8	21.8
NKPS	46.9	49.2	30.0	38.4	44.4	30.2	41.2
WKPS	2.70	2.10	1.6	1.1	2.0	1.10	1.70
TKW	43.7	41.9	38.8	42.1	34.7	33.2	37.4
GY	4.30	5.20	3.3	4.3	3.7	3.10	4.00

**Table 9:** Eigenvalues, eigenvectors, and the total percentage of variation explained by the first three principal components of barley accessions evaluated for 11 quantitative traits.

PCAs	DTH	DTM	PLHT	PDL	NFT	SL	NSPS	NKPS	WKPS	TKW	GY	Eig. v	Var. ex (%)	Cum. var. ex (%)
PC1	0.28	0.30	0.28	0.07	0.32	−0.10	0.37	0.38	0.34	0.31	0.40	5.26	47.79	47.79
PC2	−0.37	−0.28	0.03	0.38	0.27	0.46	0.26	−0.14	−0.32	0.39	0.16	2.26	20.54	68.33
PC3	0.17	−0.01	0.60	0.63	−0.33	0.12	−0.14	−0.03	0.13	−0.09	−0.24	1.25	11.33	79.65

*Note:* Cum. var. ex, cumulative variance explained in percentage; Var. ex, variance explained in percentage.

Abbreviations: DTH, days to heading; DTM, days to maturity; Eig. v, eigenvalue; NFT, number of fertile tillers; NKPS, number of kernels per spike; NSPS, number of spikelets per spike; PLHT, plant height; TKW, thousand kernel weight; WKPS, weight of kernels per spike.

## Data Availability

All relevant data are within the manuscript and its Supporting Information.

## Supporting Information

Supporting Information 1 Table S1: List and description of barley accessions used in the experiment.

Supporting Information 2 Table S2: Mean performance of 49 barley accessions for 11 morphoagronomic traits.

Supporting Information 3 Table S3: Euclidean distances of 49 barley accessions based on 11 morphoagronomic traits.
