# Supplementary material for: Genetic Variability and Association of Morpho-Agronomic Traits Among Ethiopian Barley (Hordeum vulgare L) Accessions
Source: Scientifica (Cairo). 2025 Feb 7;2025:3957883. doi: 10.1155/sci5/3957883 (PMC11828655; doi:10.1155/sci5/3957883)
Supplement: Supporting Information 1 — Table S1: List and description of barley accessions used in the experiment. [file 3957883.f1.docx]

Table S1. List and description of barley accessions used in the experiment

| Accession code | Collection area | | Coordinate | | Altitude (m.a.s.l) |
| --- | --- | --- | --- | --- | --- |
|  | region | Geographic origin | Latitude | Longitude |  |
| 4423 | Amhara | Gojjam | 10-42-00-N | 37-34-00-E | 2500 |
| 4425 |  |  | 10-43-00-N | 37-35-00-E | 2450 |
| 4426 |  |  | 10-43-00-N | 37-35-00-E | 2450 |
| 4427 |  |  | 10-43-00-N | 37-35-00-E | 2450 |
| 212737 |  |  | 37-48-00-N | 10-23-00-E | 2520 |
| 9950 |  |  | 10-18-82-N | 37-42-15-E | 2360 |
| 9949 |  |  | 10-26-98-N | 37-44-03-E | 2607 |
| 4366 |  |  | 10-21-00-N | 37-34-00-E | 2410 |
| 243286 |  | Gonder | 11-48-09-N | 38-09-51-E | 2797 |
| 243287 |  |  | 11-48-09-N | 38-09-51-E | 2797 |
| 243288 |  |  | 11-47-51-N | 38-08-04-E | 2805 |
| 243289 |  |  | 11-47-51-N | 38-08-04-E | 2805 |
| 243597 |  |  | 13-08-00-N | 37-56-00-E | 3120 |
| 243598 |  |  | 13-08-00-N | 37-56-00-E | 3120 |
| 243599 |  |  | 13-08-00-N | 37-56-00-E | 3120 |
| 243600 |  |  | 13-08-00-N | 37-56-00-E | 3115 |
| 243568 |  | Wollo | 11-50-00-N | 39-33-00-E | 2330 |
| 243571 |  |  | 11-51-00-N | 39-30-00-E | 2980 |
| 243572 |  |  | 11-51-00-N | 39-30-00-E | 2980 |
| 235066 |  |  | 11-49-00-N | 39-31-00-E | 3010 |
| 235072 |  |  | 11-11-00-N | 39-30-00-E | 2830 |
| 235073 |  |  | 11-13-00-N | 39-31-00-E | 2950 |
| 235074 |  |  | 11-13-00-N | 39-32-00-E | 2950 |
| 235075 |  |  | 11-14-00-N | 39-28-00-E | 3150 |
| 8525 |  | North Shewa | 09-43-39-N | 39-45-33-E | 2338 |
| 8526 |  |  | 09-34-40-N | 39-44-43-E | 2478 |
| 8556 |  |  | 09-34-39-N | 39-44-50-E | 2388 |
| 8557 |  |  | 09-34-46-N | 39-44-30-E | 2452 |
| 8558 |  |  | 09-55-08-N | 39-44-06-E | 2518 |
| 243232 | Oromia |  | 09-34-34-N | 39-29-29-E | 2801 |
| 243231 |  |  | 09-34-34-N | 39-29-29-E | 2801 |
| 243230 |  |  | 09-34-34-N | 39-29-29-E | 2801 |
| 243229 |  |  | 09-34-34-N | 39-29-29-E | 2801 |
| 232219 |  | Arsi | 08-28-00-N | 39-27-00-E | 2360 |
| 232220 |  |  | 08-28-00-N | 39-37-00-E | 2360 |
| 232221 |  |  | 08-27-00-N | 39-36-00-E | 2550 |
| 232222 |  |  | 08-26-00-N | 39-37-00-E | 2750 |
| 237002 |  |  | 07-19-00-N | 39-16-00-E | 2360 |
| 237003 |  |  | 07-19-00-N | 39-16-00-E | 2350 |
| 237004 |  |  | 07-19-00-N | 39-16-00-E | 2670 |
| 237011 |  |  | 07-19-00-N | 39-16-00-E | 2790 |
| 243191 |  | Bale | 07-16-42-N | 39-51-16-E | 2557 |
| 243192 |  |  | 07-15-55-N | 39-52-33-E | 2470 |
| 243193 |  |  | 07-15-22-N | 39-49-40-E | 2500 |
| 243195 |  |  | 07-15-50-N | 39-58-24-E | 2510 |
| 243213 |  |  | 07-02-30-N | 39-30-45-E | 2621 |
| 243214 |  |  | 07-02-30-N | 39-32-18-E | 2852 |
| 243215 |  |  | 07-02-11-N | 39-32-18-E | 2852 |
| 243216 |  |  | 07-02-11-N | 39-32-18-E | 2852 |
