# Supplementary material for: Genetic Variability and Association of Morpho-Agronomic Traits Among Ethiopian Barley (Hordeum vulgare L) Accessions
Source: Scientifica (Cairo). 2025 Feb 7;2025:3957883. doi: 10.1155/sci5/3957883 (PMC11828655; doi:10.1155/sci5/3957883)
Supplement: Supporting Information 2 — Table S2: Mean performance of 49 barley accessions for 11 morphoagronomic traits. [file 3957883.f2.docx]

Table S2. Mean values of 49 barley accessions for 11 morpho-agronomic traits.

| Accession | DTH | DTM | PLHT | PDL | NFT | SL | NSPS | NKPS | WKPS | TKW | GY |
| --- | --- | --- | --- | --- | --- | --- | --- | --- | --- | --- | --- |
| 4366 | 61.5^tuv^ | 98^x^ | 100.8^d-m^ | 40.5^a-d^ | 3.37^a-i^ | 9.07^a-d^ | 21.8^f-o^ | 32.6^m-r^ | 1.01^p-s^ | 35.52^e-m^ | 3.30^q-x^ |
| 4423 | 67 ^o-s^ | 125.5^abc^ | 126.4^a^ | 37.9^c-i^ | 2.27^i^ | 8.5^a-h^ | 20.87^j-o^ | 30.04^o-s^ | 1.58^j-p^ | 38.84^e-g^ | 3.33^p-x^ |
| 4425 | 64.5^r-v^ | 102^u-x^ | 94.5^i-p^ | 35.4^f-m^ | 2.82^b-i^ | 7.64^f-o^ | 19.59^nop^ | 31.22^n-s^ | 1.02^p-s^ | 31.58^k-o^ | 2.92^u-y^ |
| 4426 | 60^v^ | 102.5^t-x^ | 99.84^e-n^ | 41.9^ab^ | 3.4^a-h^ | 9.27^abc^ | 22.41^c-m^ | 38.10^g-o^ | 1.13^n-s^ | 45.64^abc^ | 4.38^f-n^ |
| 4427 | 60.5^uv^ | 105.5^r-v^ | 92.34^k-q^ | 38.4^c-g^ | 3.98^a^ | 8.77^a-f^ | 24.8^a-d^ | 48.60^a-g^ | 0.95^qrs^ | 48.7^a^ | 5.99^ab^ |
| 8525 | 76^e-j^ | 113^i-o^ | 99.04^e-n^ | 39.3^b-e^ | 2.78^b-i^ | 8.70^a-g^ | 21.1^i-o^ | 44.85^b-l^ | 1.94^e-k^ | 36.31^e-l^ | 3.57^l-v^ |
| 8526 | 83.5^ab^ | 121^b-g^ | 116.93^b^ | 38.34^c-g^ | 3.30^a-i^ | 7^k-q^ | 22.8^b-l^ | 49.3^a-f^ | 2.22^a-h^ | 46.35^ab^ | 5.21^a-g^ |
| 8556 | 76.5^d-i^ | 110.5^m-r^ | 103.82^c-h^ | 37.25^d-j^ | 3.95^a^ | 5.93^qr^ | 24.64^a-e^ | 53.39^abc^ | 2.4^a-e^ | 45.37^abc^ | 5.8^abc^ |
| 8557 | 79^b-f^ | 114.5^h-n^ | 106.3^c- f^ | 38.17^c-h^ | 2.93^a-i^ | 6.17^pqr^ | 19.74^m-p^ | 39.67^e-o^ | 1.92^e-k^ | 35.42^e-m^ | 3.08^t-y^ |
| 8558 | 76.5^d-i^ | 112.5^j-p^ | 109.04^bcd^ | 37.84^c-i^ | 2.35^ghi^ | 7.07^j-q^ | 20.4^l-o^ | 45.7^a-k^ | 2.52^a-d^ | 34.27^g-m^ | 3.12^s-y^ |
| 9949 | 60.5^uv^ | 104^s-w^ | 97.64^f-n^ | 43.34^a^ | 3.34^a-i^ | 8.3^a-j^ | 21.57^g-o^ | 46.02^a-k^ | 1.03^o-s^ | 43.1^bcd^ | 4.27^g-q^ |
| 9950 | 61.5^tuv^ | 107^p-u^ | 88.1^opq^ | 37.7^c-i^ | 3.17^a-i^ | 8.43^a-h^ | 19.31^op^ | 26.46^p-s^ | 0.85^rs^ | 40.16^def^ | 3.58^a-v^ |
| 212737 | 80.5^a-e^ | 118.5^d-i^ | 109.6^bcd^ | 37.7^c-i^ | 3.76^ab^ | 8.80^a-f^ | 24.3^a-f^ | 44.87^b-l^ | 1.88^e-l^ | 44.07^a-d^ | 5.33^a-e^ |
| 232219 | 72.5^i-n^ | 112^k-q^ | 107.84^cde^ | 40.84^abc^ | 2.87^a-i^ | 6.87^m-r^ | 22.17^d-n^ | 52.57^a-d^ | 2.31^a-g^ | 32.75^i-o^ | 4.12^h-r^ |
| 232220 | 71.5^j-o^ | 108^q-t^ | 104.9^c-g^ | 36.9^e-k^ | 2.6^c-i^ | 7^k-q^ | 21.2^i-o^ | 39.5^e-o^ | 1.82^f-l^ | 32.17^j-o^ | 3.17^r-y^ |
| 232221 | 74^g-m^ | 109^n-s^ | 101.9^d-j^ | 36.6^e-k^ | 2.38^f-i^ | 7.77^e-n^ | 19.63^nop^ | 38.98^j-p^ | 1.76^f-l^ | 30.26^mno^ | 2.39^xy^ |
| 232222 | 76.5^d-i^ | 115.5^g-m^ | 99.17^e-n^ | 37.33^d-j^ | 3.07^a-i^ | 8.37^a-i^ | 21.09^i-o^ | 46.4^a-j^ | 1.76^f-l^ | 34.38^g-m^ | 4.07^i-s^ |
| 235066 | 71^k-o^ | 113^i-o^ | 95.64^h-o^ | 37.23^d-j^ | 3.07^a-i^ | 7.14^i-q^ | 19.4^op^ | 42.53^c-m^ | 1.77^f-l^ | 33.93^h-m^ | 3.94^j-t^ |
| 235072 | 74^g-m^ | 117.5^e-k^ | 94^j-p^ | 34.87^h-o^ | 2.3^hi^ | 8.13^a-l^ | 19.48^nop^ | 34.7^l-q^ | 1.6^j-o^ | 30.99^l-o^ | 3.38^o-w^ |
| 235073 | 76^e-j^ | 116^g-m^ | 91.73^m-q^ | 38.07^c-i^ | 2.52^e-i^ | 8.80^a-f^ | 20.47^k-o^ | 37.1^h-p^ | 1.72^g-l^ | 33.39^i-m^ | 2.89^u-y^ |
| 235074 | 71^k-o^ | 111^l-r^ | 84.53^qrs^ | 34.83^h-o^ | 2.55^d-i^ | 8.2^a-k^ | 19.74^m-p^ | 36.3^i-p^ | 1.48^k-q^ | 31.39^k-o^ | 2.96^t-y^ |
| 235075 | 71^k-o^ | 111.5^l-q^ | 95.87^g-o^ | 36.97^e-k^ | 2.74^b-i^ | 8.97^a-e^ | 21.2^i-o^ | 44^b-l^ | 1.78^f-l^ | 32.83^i-o^ | 3.64^k-v^ |
| 237002 | 68.5^n-r^ | 106.5^q-u^ | 103.57^c-i^ | 41^abc^ | 2.92^a-i^ | 9.34^ab^ | 23.14^a-k^ | 29.8^o-s^ | 1.12^n-s^ | 38.91^e-h^ | 3.5^m-w^ |
| 237003 | 65^q-u^ | 113^i-o^ | 94.4^j-p^ | 37.4^d-j^ | 3.39^a-i^ | 7.3^h-p^ | 20.84^j-o^ | 41.74^d-n^ | 1.63^i-n^ | 39.58^d-g^ | 4.49^e-l^ |
| 237004 | 66^p-t^ | 109^n-s^ | 93.64^j-p^ | 38.34^c-g^ | 2.9^a-i^ | 9.14^a-d^ | 21.24^h-o^ | 29.2^o-s^ | 1.14^n-s^ | 37.55^e-j^ | 3.15^r-y^ |
| 237011 | 82^abc^ | 125a^bc^ | 104.8^c-g^ | 34.87^h-o^ | 3.45^a-g^ | 7.63^f-o^ | 23.17^a-k^ | 49.97^a-e^ | 2.2^a-i^ | 39.63^e-g^ | 5.08^b-h^ |
| 243191 | 64.5^r-v^ | 108^q-t^ | 86.57^p-s^ | 36.3^e-k^ | 2.98^a-i^ | 8.84^a-f^ | 19.72^m-p^ | 27.11^p-s^ | 1.06^n-s^ | 33.13^i-n^ | 2.55^wxy^ |
| 243192 | 65.5^q-t^ | 108^o-t^ | 87.67^o-r^ | 34.74^i-o^ | 3.05^a-i^ | 8.14^a-l^ | 21.58^g-o^ | 30.8^o-s^ | 1.11^n-s^ | 37.80^e-i^ | 3.85^k-u^ |
| 243193 | 68.5^n-r^ | 115.5^g-m^ | 102.04^d-j^ | 35.07^g-n^ | 3.43^a-g^ | 7.97^d-m^ | 23.9^a-h^ | 42.94^b-m^ | 1.7^h-m^ | 33.014^f-m^ | 4.3^g-p^ |
| 243195 | 78^c-g^ | 122.5^b-e^ | 99.47^e-n^ | 34.8^h-o^ | 3.77^ab^ | 7.04^k-q^ | 22.84^b-l^ | 47.8^a-h^ | 1.81^f-l^ | 35.01^f-m^ | 4.61^d-k^ |
| 243213 | 77.5^c-h^ | 120.5^c-g^ | 95.8^g-o^ | 37.14^d-j^ | 3.58^a-e^ | 7.97^d-m^ | 21.4^h-o^ | 44.37^b-l^ | 1.78^f-l^ | 34.15^h-m^ | 4.17^h-q^ |
| 243214 | 81^a-d^ | 122^b-f^ | 99.9^e-n^ | 36.47^e-k^ | 3.6^abc^ | 5.74^r^ | 22.13^e-n^ | 48.47^a-g^ | 1.97^d-k^ | 40.23^def^ | 5.28^a-f^ |
| 243215 | 81^a-d^ | 124.5^abc^ | 105.13^c-f^ | 36.17^e-l^ | 3.99^a^ | 7.77^e-n^ | 25.2^ab^ | 56.43^a^ | 2.6^abc^ | 48.31^ab^ | 5.64^abc^ |
| 243216 | 77^d-i^ | 123.5^bdc^ | 99.6^e-n^ | 35.8^f-m^ | 3.65^a-d^ | 8.04^c-m^ | 22.94^b-l^ | 39.47^e-o^ | 1.71^h-l^ | 35.96^e-l^ | 4.88^c-j^ |
| 243229 | 78^c-g^ | 123^b-e^ | 103.57^c-i^ | 36.37^e-k^ | 3.7^abc^ | 7.9^d-m^ | 25.76^a^ | 52.17^a-d^ | 2.64^ab^ | 47.06^ab^ | 6.06^a^ |
| 243230 | 73^h-n^ | 116^g-m^ | 101.24^d-k^ | 32.04^no^ | 3.67^abc^ | 6.5^o-r^ | 23.42^a-j^ | 53.07^abc^ | 2.26^a-h^ | 36.8^e-k^ | 4.99_c-i_ |
| 243231 | 73.5^g-m^ | 115.5^g-m^ | 79.47^rst^ | 31.57^o^ | 2.3^hi^ | 6.9^l-r^ | 17.57^p^ | 38.28^g-o^ | 1.15^m-s^ | 27.79^o^ | 2.28y |
| 243232 | 70.5^l-p^ | 116.5^f-l^ | 92^l-q^ | 35.54^f-m^ | 3.37^a-i^ | 7.5^g-o^ | 20.9^j-o^ | 49.3^a-f^ | 2.06^c-k^ | 35.17^e-m^ | 3.86^k-u^ |
| 243286 | 62^tuv^ | 100.5^vwx^ | 79.17^rst^ | 34.17^j-o^ | 2.54^d-i^ | 7.5^g-o^ | 19.42^op^ | 21.6^s^ | 0.73^s^ | 33.01l^-s^ | 2.73v-y |
| 243287 | 64^r-v^ | 104s-w | 72.3^t^ | 32.87^l-o^ | 2.72^b-i^ | 8.24^a-k^ | 19.67^nop^ | 23.53^rs^ | 0.82^rs^ | 32.87l-o | 2.97t-y |
| 243288 | 63^s-v^ | 100wx | 78.57^st^ | 34.84^h-o^ | 2.75^b-i^ | 8.63^a-g^ | 20.03^m-p^ | 24.83^rs^ | 0.86^rs^ | 33.51^h-m^ | 2.83v-y |
| 243289 | 61.5^tuv^ | 126.5^ab^ | 92.27^k-q^ | 32.57^mno^ | 3.1^a-i^ | 8.2^a-k^ | 23.51a-j | 46.94^a-i^ | 2.7^a^ | 43.7^a-d^ | 4.33^f-o^ |
| 243568 | 69.5^m-q^ | 112^k-q^ | 109.8^bcd^ | 37.37^d-j^ | 3.2^a-i^ | 8.1^b-m^ | 23.71^a-i^ | 52.56^a-d^ | 2.09^b-j^ | 40.11^def^ | 4.22^h-q^ |
| 243571 | 75^f-l^ | 111^l-r^ | 101.03^d-l^ | 40.5^a-d^ | 3.2^a-i^ | 6.57^n-r^ | 20.87^j-o^ | 42.5^c-m^ | 2.34^a-f^ | 34.28^g-m^ | 3.6^l-v^ |
| 243572 | 75^f-l^ | 114.5^h-n^ | 94.4^j-p^ | 36.2^e-l^ | 3.24^a-i^ | 6.64^n-r^ | 21.5^h-o^ | 53.54^ab^ | 2.17^a-j^ | 35.27^e-m^ | 4.45^e-m^ |
| 243597 | 75.5^f-k^ | 117.5^e-k^ | 90.73^n-q^ | 35.94^e-l^ | 3.5^a-f^ | 8.04^c-m^ | 25.47^ab^ | 53.4^abc^ | 2.11^b-j^ | 45.56^abc^ | 5.45^a-d^ |
| 243598 | 74.5^f-l^ | 120^c-h^ | 92.9^j-q^ | 39.27^b-e^ | 3.57^a-e^ | 8.93^a-e^ | 24.2^a-g^ | 35.25^k-q^ | 1.34^l-r^ | 40.53^cde^ | 4.45^f-o^ |
| 243599 | 85^a^ | 129^a^ | 111.7^4bc^ | 38.73^b-f^ | 2.43^d-i^ | 9.37^a^ | 24.9^abc^ | 46.47^a-j^ | 2.14^a-j^ | 45.78a^b^ | 5.63^abc^ |
| 243600 | 75.5^f-k^ | 118^d-j^ | 85.6^p-s^ | 33.7^k-o^ | 2.28^hi^ | 6.94^l-q^ | 20.05^m-p^ | 38.48^f-o^ | 1.7^h-m^ | 27.99^no^ | 3.47^n-w^ |

DTH= days to heading, DTM = days to maturity, PLHT = plant height, NFT = number of fertile tillers, NSPS= number of spiklet per spike,

NKPS= number of kernels per spike, WKPS= weight of kernels per spike, TKW= thousand kernel weight.
