# Supplementary material for: Genetic Variability and Association of Morpho-Agronomic Traits Among Ethiopian Barley (Hordeum vulgare L) Accessions
Source: Scientifica (Cairo). 2025 Feb 7;2025:3957883. doi: 10.1155/sci5/3957883 (PMC11828655; doi:10.1155/sci5/3957883)
Supplement: Supporting Information 3 — Table S3: Euclidean distances of 49 barley accessions based on 11 morphoagronomic traits. [file 3957883.f3.docx]

Table S3. Euclidean distances of 49 barley accessions based on 11 morpho-agronomic traits.

|  | 4423 | 4425 | 4426 | 4427 | 8525 | 8526 | 8556 | 8557 | 8558 | 9949 | 9950 | 212737 | 232219 | 232220 | 232221 | 232222 | 235066 | 235072 | 235073 | 235074 |
| --- | --- | --- | --- | --- | --- | --- | --- | --- | --- | --- | --- | --- | --- | --- | --- | --- | --- | --- | --- | --- |
| 4366 | 5.34 | 3.26 | 2.42 | 4.74 | 3.90 | 6.63 | 6.74 | 5.25 | 5.45 | 2.79 | 2.80 | 5.51 | 4.96 | 4.10 | 4.35 | 4.21 | 4.20 | 4.96 | 4.21 | 4.37 |
| 4423 |  | 5.13 | 5.52 | 7.15 | 4.07 | 5.12 | 7.09 | 4.42 | 4.12 | 5.69 | 5.20 | 5.26 | 4.84 | 4.04 | 4.11 | 4.27 | 4.57 | 4.11 | 4.16 | 5.08 |
| 4425 |  |  | 4.84 | 6.24 | 3.98 | 6.76 | 6.93 | 4.07 | 4.48 | 4.72 | 2.53 | 6.33 | 4.97 | 2.75 | 2.62 | 3.85 | 2.97 | 3.02 | 3.44 | 2.26 |
| 4426 |  |  |  | 3.19 | 4.16 | 5.92 | 6.04 | 5.85 | 5.96 | 1.65 | 3.42 | 4.74 | 5.15 | 5.08 | 5.63 | 4.53 | 4.77 | 5.86 | 4.96 | 5.46 |
| 4427 |  |  |  |  | 5.54 | 5.86 | 4.97 | 6.94 | 7.22 | 3.60 | 5.01 | 4.37 | 6.06 | 6.32 | 7.25 | 5.28 | 5.65 | 7.00 | 6.46 | 6.56 |
| 8525 |  |  |  |  |  | 4.16 | 5.31 | 3.09 | 2.61 | 3.95 | 4.11 | 3.77 | 2.75 | 2.60 | 2.74 | 1.33 | 2.41 | 2.92 | 1.72 | 3.12 |
| 8526 |  |  |  |  |  |  | 3.08 | 3.99 | 4.22 | 5.71 | 6.65 | 2.63 | 3.77 | 4.79 | 5.76 | 3.85 | 4.50 | 5.72 | 5.39 | 6.34 |
| 8556 |  |  |  |  |  |  |  | 4.99 | 5.39 | 5.72 | 6.89 | 3.69 | 4.27 | 5.36 | 6.71 | 4.79 | 4.90 | 6.76 | 6.52 | 6.83 |
| 8557 |  |  |  |  |  |  |  |  | 2.11 | 5.27 | 4.91 | 4.96 | 2.85 | 2.11 | 2.74 | 2.92 | 2.20 | 3.39 | 3.40 | 3.85 |
| 8558 |  |  |  |  |  |  |  |  |  | 5.54 | 5.50 | 5.10 | 2.36 | 1.99 | 2.45 | 2.87 | 2.76 | 3.25 | 3.15 | 3.86 |
| 9949 |  |  |  |  |  |  |  |  |  |  | 3.63 | 5.04 | 4.40 | 4.71 | 5.39 | 4.30 | 4.23 | 5.73 | 4.85 | 5.32 |
| 9950 |  |  |  |  |  |  |  |  |  |  |  | 5.82 | 5.60 | 4.15 | 4.15 | 4.15 | 3.55 | 3.97 | 3.78 | 3.30 |
| 212737 |  |  |  |  |  |  |  |  |  |  |  |  | 4.42 | 5.05 | 5.82 | 3.35 | 4.61 | 5.56 | 4.93 | 5.90 |
| 232219 |  |  |  |  |  |  |  |  |  |  |  |  |  | 2.69 | 3.81 | 2.82 | 2.85 | 4.42 | 3.93 | 4.71 |
| 232220 |  |  |  |  |  |  |  |  |  |  |  |  |  |  | 1.63 | 2.55 | 1.97 | 2.59 | 2.76 | 2.78 |
| 232221 |  |  |  |  |  |  |  |  |  |  |  |  |  |  |  | 2.94 | 2.59 | 1.90 | 2.10 | 2.16 |
| 232222 |  |  |  |  |  |  |  |  |  |  |  |  |  |  |  |  | 1.89 | 2.69 | 2.16 | 2.97 |
| 235066 |  |  |  |  |  |  |  |  |  |  |  |  |  |  |  |  |  | 2.56 | 2.68 | 2.58 |
| 235072 |  |  |  |  |  |  |  |  |  |  |  |  |  |  |  |  |  |  | 1.83 | 1.52 |
| 235073 |  |  |  |  |  |  |  |  |  |  |  |  |  |  |  |  |  |  |  | 2.02 |

|  | 235075 | 237002 | 237003 | 237004 | 237011 | 243191 | 243192 | 243193 | 243195 | 243213 | 243214 | 243215 | 243216 | 243229 | 243230 | 243231 | 243232 |
| --- | --- | --- | --- | --- | --- | --- | --- | --- | --- | --- | --- | --- | --- | --- | --- | --- | --- |
| 4366 | 3.62 | 2.09 | 3.77 | 2.27 | 6.49 | 3.12 | 3.34 | 4.20 | 5.83 | 4.74 | 6.70 | 7.52 | 5.17 | 7.16 | 6.49 | 6.64 | 4.79 |
| 4423 | 4.24 | 4.20 | 4.76 | 4.27 | 5.29 | 5.16 | 5.05 | 4.54 | 5.50 | 4.86 | 6.15 | 6.86 | 4.80 | 6.53 | 6.27 | 6.56 | 5.13 |
| 4425 | 3.20 | 3.93 | 3.44 | 2.64 | 6.13 | 1.89 | 2.21 | 4.01 | 5.20 | 4.39 | 6.06 | 7.91 | 4.96 | 7.60 | 5.63 | 3.72 | 3.90 |
| 4426 | 4.28 | 2.51 | 3.66 | 3.16 | 6.17 | 4.54 | 4.01 | 4.46 | 5.92 | 4.98 | 6.41 | 6.55 | 5.16 | 6.16 | 6.49 | 7.80 | 5.06 |
| 4427 | 5.44 | 4.81 | 4.10 | 5.09 | 5.69 | 6.14 | 4.70 | 4.42 | 5.43 | 5.27 | 5.84 | 5.40 | 4.97 | 5.03 | 5.60 | 8.57 | 5.27 |
| 8525 | 1.47 | 3.08 | 2.98 | 2.92 | 3.79 | 3.84 | 3.62 | 3.10 | 3.79 | 2.42 | 4.54 | 5.34 | 3.29 | 5.07 | 4.74 | 5.33 | 2.69 |
| 8526 | 5.04 | 5.63 | 4.31 | 6.04 | 2.42 | 7.10 | 5.92 | 4.16 | 3.44 | 3.78 | 2.81 | 2.84 | 3.54 | 2.90 | 3.98 | 7.77 | 4.31 |
| 8556 | 5.76 | 6.43 | 4.19 | 6.68 | 3.47 | 7.49 | 5.93 | 4.19 | 3.56 | 4.43 | 2.68 | 2.94 | 4.18 | 2.89 | 3.09 | 8.23 | 4.35 |
| 8557 | 3.67 | 4.84 | 3.36 | 4.48 | 4.08 | 4.77 | 4.40 | 3.90 | 3.58 | 3.12 | 3.53 | 5.90 | 3.89 | 5.84 | 4.39 | 4.79 | 3.12 |
| 8558 | 3.12 | 4.88 | 3.91 | 4.64 | 4.20 | 5.10 | 4.81 | 4.00 | 4.25 | 3.67 | 4.50 | 5.96 | 4.40 | 5.74 | 4.58 | 5.16 | 3.29 |
| 9949 | 4.23 | 3.11 | 3.30 | 3.56 | 6.08 | 4.71 | 4.26 | 4.52 | 5.64 | 4.71 | 5.90 | 6.58 | 5.19 | 6.28 | 6.28 | 7.42 | 4.69 |
| 9950 | 3.68 | 3.26 | 3.05 | 1.87 | 6.23 | 1.95 | 2.01 | 4.30 | 5.50 | 4.45 | 6.16 | 7.54 | 4.82 | 7.20 | 6.22 | 5.15 | 4.28 |
| 212737 | 4.31 | 4.50 | 3.99 | 5.02 | 2.45 | 6.21 | 5.02 | 3.19 | 3.21 | 3.15 | 3.79 | 2.64 | 2.51 | 2.46 | 4.09 | 7.83 | 4.09 |
| 232219 | 3.37 | 4.59 | 3.38 | 4.82 | 3.96 | 5.57 | 5.00 | 3.52 | 3.77 | 3.31 | 3.88 | 5.22 | 4.00 | 4.96 | 4.22 | 6.33 | 3.16 |
| 232220 | 2.46 | 3.89 | 2.99 | 3.42 | 4.45 | 3.70 | 3.31 | 3.05 | 3.88 | 3.29 | 4.48 | 6.31 | 3.92 | 6.01 | 4.28 | 4.34 | 2.85 |
| 232221 | 2.47 | 4.10 | 3.90 | 3.27 | 5.24 | 3.16 | 3.59 | 4.00 | 4.76 | 3.78 | 5.56 | 7.27 | 4.59 | 7.02 | 5.40 | 3.65 | 3.51 |
| 232222 | 1.50 | 3.63 | 2.51 | 3.23 | 2.93 | 3.88 | 3.30 | 2.29 | 2.67 | 1.39 | 3.72 | 4.82 | 2.29 | 4.61 | 3.77 | 4.92 | 1.88 |
| 235066 | 2.32 | 4.19 | 1.80 | 3.35 | 3.88 | 3.49 | 2.96 | 2.86 | 3.10 | 2.22 | 3.62 | 5.72 | 3.16 | 5.50 | 3.82 | 4.11 | 1.68 |
| 235072 | 2.30 | 4.44 | 3.69 | 3.26 | 4.71 | 3.06 | 3.12 | 3.73 | 4.31 | 3.36 | 5.24 | 6.93 | 3.92 | 6.61 | 5.10 | 3.00 | 3.20 |
| 235073 | 1.68 | 3.41 | 3.57 | 2.55 | 4.62 | 2.92 | 3.28 | 3.66 | 4.36 | 2.95 | 5.30 | 6.49 | 3.78 | 6.21 | 5.44 | 4.20 | 3.15 |
| 235074 | 2.24 | 4.29 | 3.48 | 2.80 | 5.26 | 2.14 | 2.42 | 3.77 | 4.58 | 3.56 | 5.57 | 7.24 | 4.29 | 6.93 | 5.22 | 2.68 | 3.07 |
| 235075 |  | 3.20 | 2.87 | 2.49 | 4.12 | 3.05 | 2.84 | 2.64 | 3.83 | 2.60 | 4.98 | 5.86 | 3.31 | 5.50 | 4.54 | 4.63 | 2.43 |
| 237002 |  |  | 3.82 | 1.88 | 5.65 | 3.50 | 3.41 | 3.86 | 5.40 | 4.27 | 6.21 | 6.71 | 4.46 | 6.29 | 6.33 | 6.71 | 4.72 |
| 237003 |  |  |  | 3.16 | 3.83 | 3.71 | 2.51 | 2.36 | 3.08 | 2.52 | 3.52 | 5.06 | 2.91 | 4.75 | 3.59 | 5.26 | 1.97 |
| 237004 |  |  |  |  | 5.55 | 1.74 | 2.07 | 3.57 | 5.05 | 3.81 | 6.01 | 6.96 | 4.20 | 6.58 | 5.93 | 5.20 | 3.90 |
| 237011 |  |  |  |  |  | 6.27 | 5.04 | 2.94 | 1.76 | 2.50 | 2.41 | 2.55 | 2.00 | 2.53 | 2.58 | 6.58 | 3.06 |
| 243191 |  |  |  |  |  |  | 2.12 | 4.21 | 5.46 | 4.29 | 6.53 | 7.93 | 4.85 | 7.62 | 6.24 | 4.10 | 4.08 |
| 243192 |  |  |  |  |  |  |  | 2.94 | 4.27 | 3.59 | 5.20 | 6.54 | 3.73 | 6.14 | 4.76 | 4.40 | 3.24 |
| 243193 |  |  |  |  |  |  |  |  | 2.25 | 2.25 | 3.72 | 4.43 | 1.94 | 4.05 | 2.77 | 5.61 | 2.20 |
| 243195 |  |  |  |  |  |  |  |  |  | 1.77 | 2.06 | 3.63 | 1.55 | 3.63 | 2.07 | 5.72 | 2.27 |
| 243213 |  |  |  |  |  |  |  |  |  |  | 3.03 | 4.35 | 1.47 | 4.26 | 3.39 | 5.20 | 1.76 |
| 243214 |  |  |  |  |  |  |  |  |  |  |  | 3.56 | 2.95 | 3.63 | 2.72 | 6.47 | 3.28 |
| 243215 |  |  |  |  |  |  |  |  |  |  |  |  | 3.79 | 1.07 | 3.75 | 8.76 | 4.71 |
| 243216 |  |  |  |  |  |  |  |  |  |  |  |  |  | 3.56 | 3.12 | 5.91 | 2.59 |
| 243229 |  |  |  |  |  |  |  |  |  |  |  |  |  |  | 3.65 | 8.60 | 4.57 |
| 243230 |  |  |  |  |  |  |  |  |  |  |  |  |  |  |  | 6.19 | 2.79 |
| 243231 |  |  |  |  |  |  |  |  |  |  |  |  |  |  |  |  | 4.58 |

|  | 243286 | 243287 | 243288 | 243289 | 243568 | 243571 | 243572 | 243597 | 243598 | 243599 | 243600 |
| --- | --- | --- | --- | --- | --- | --- | --- | --- | --- | --- | --- |
| 4366 | 4.54 | 4.83 | 3.73 | 6.53 | 4.43 | 4.67 | 5.49 | 6.11 | 4.11 | 6.96 | 5.99 |
| 4423 | 6.50 | 6.78 | 6.32 | 5.56 | 4.44 | 5.00 | 5.61 | 6.42 | 4.97 | 5.58 | 5.43 |
| 4425 | 2.14 | 2.70 | 2.14 | 6.03 | 4.89 | 4.36 | 4.62 | 6.40 | 5.01 | 7.74 | 3.62 |
| 4426 | 5.91 | 6.04 | 5.11 | 6.03 | 4.09 | 5.08 | 5.61 | 5.21 | 3.69 | 6.01 | 6.90 |
| 4427 | 7.12 | 6.92 | 6.41 | 5.60 | 4.38 | 6.19 | 5.56 | 3.94 | 4.05 | 5.76 | 7.55 |
| 8525 | 5.50 | 5.44 | 4.88 | 4.72 | 2.70 | 2.68 | 3.10 | 4.23 | 3.17 | 4.61 | 3.91 |
| 8526 | 8.27 | 8.30 | 7.99 | 5.10 | 3.22 | 3.97 | 3.67 | 3.58 | 4.44 | 3.08 | 6.12 |
| 8556 | 8.25 | 8.26 | 8.01 | 5.14 | 3.63 | 4.32 | 3.46 | 3.06 | 5.05 | 4.91 | 6.66 |
| 8557 | 5.59 | 5.94 | 5.68 | 5.59 | 3.89 | 1.88 | 2.86 | 5.37 | 4.74 | 5.94 | 3.54 |
| 8558 | 6.06 | 6.33 | 5.96 | 5.13 | 3.42 | 2.38 | 3.06 | 5.34 | 5.19 | 5.82 | 3.62 |
| 9949 | 5.92 | 6.19 | 5.34 | 6.23 | 4.01 | 4.42 | 5.03 | 5.27 | 3.99 | 6.30 | 6.53 |
| 9950 | 2.99 | 3.11 | 2.47 | 5.80 | 5.05 | 4.87 | 5.20 | 5.99 | 4.08 | 7.15 | 4.91 |
| 212737 | 7.76 | 7.51 | 7.11 | 4.66 | 2.81 | 4.55 | 4.08 | 2.64 | 2.78 | 1.98 | 6.26 |
| 232219 | 6.69 | 6.95 | 6.45 | 5.24 | 2.65 | 1.78 | 2.57 | 4.57 | 4.57 | 5.31 | 4.62 |
| 232220 | 4.45 | 4.85 | 4.42 | 5.09 | 3.26 | 2.41 | 2.96 | 5.21 | 4.50 | 6.21 | 2.98 |
| 232221 | 4.13 | 4.43 | 4.01 | 5.72 | 4.33 | 3.33 | 3.98 | 6.15 | 4.98 | 6.80 | 2.77 |
| 232222 | 5.44 | 5.26 | 4.89 | 4.32 | 2.50 | 2.73 | 2.34 | 3.75 | 2.97 | 4.39 | 3.44 |
| 235066 | 4.52 | 4.61 | 4.36 | 4.58 | 3.30 | 2.16 | 2.11 | 4.60 | 3.94 | 5.82 | 2.87 |
| 235072 | 4.03 | 3.89 | 3.87 | 4.98 | 4.54 | 4.02 | 3.86 | 5.67 | 4.55 | 6.33 | 1.79 |
| 235073 | 4.57 | 4.41 | 4.02 | 5.06 | 4.03 | 3.49 | 3.87 | 5.21 | 3.62 | 5.64 | 3.03 |
| 235074 | 3.04 | 2.77 | 2.70 | 5.10 | 4.67 | 4.15 | 3.93 | 5.65 | 4.53 | 6.93 | 2.13 |
| 235075 | 4.69 | 4.49 | 3.98 | 4.38 | 2.94 | 3.38 | 3.22 | 4.43 | 3.43 | 5.31 | 3.33 |
| 237002 | 5.11 | 5.27 | 4.33 | 6.01 | 3.91 | 4.44 | 5.30 | 5.39 | 3.01 | 5.57 | 5.66 |
| 237003 | 4.75 | 4.78 | 4.45 | 3.91 | 2.83 | 2.91 | 2.54 | 3.74 | 3.23 | 5.41 | 4.11 |
| 237004 | 3.62 | 3.61 | 2.78 | 5.35 | 4.16 | 4.34 | 4.83 | 5.43 | 3.19 | 6.16 | 4.51 |
| 237011 | 7.53 | 7.23 | 7.15 | 3.79 | 3.00 | 4.07 | 2.75 | 2.66 | 3.79 | 3.06 | 4.89 |
| 243191 | 2.46 | 2.33 | 1.64 | 5.82 | 5.20 | 4.85 | 5.17 | 6.36 | 4.37 | 7.43 | 4.04 |
| 243192 | 2.88 | 2.67 | 2.37 | 4.63 | 4.13 | 4.43 | 4.10 | 4.79 | 3.51 | 6.41 | 3.76 |
| 243193 | 5.48 | 5.30 | 5.00 | 3.44 | 2.11 | 3.59 | 2.75 | 3.18 | 2.87 | 4.60 | 4.15 |
| 243195 | 6.59 | 6.33 | 6.31 | 4.04 | 3.16 | 3.57 | 2.14 | 3.14 | 3.56 | 4.35 | 4.22 |
| 243213 | 5.84 | 5.55 | 5.36 | 4.23 | 2.98 | 2.87 | 2.27 | 3.52 | 2.69 | 4.24 | 3.75 |
| 243214 | 7.30 | 7.24 | 7.24 | 4.84 | 3.90 | 3.54 | 2.39 | 3.49 | 4.47 | 4.74 | 4.93 |
| 243215 | 9.24 | 8.93 | 8.73 | 4.43 | 3.82 | 5.43 | 4.34 | 2.41 | 4.65 | 2.93 | 7.14 |
| 243216 | 6.27 | 5.93 | 5.81 | 3.90 | 3.08 | 3.75 | 2.94 | 3.07 | 2.42 | 3.60 | 4.33 |
| 243229 | 8.85 | 8.55 | 8.34 | 4.02 | 3.52 | 5.26 | 4.21 | 1.98 | 4.32 | 2.78 | 6.84 |
| 243230 | 6.95 | 6.75 | 6.77 | 3.66 | 3.29 | 4.27 | 2.35 | 3.24 | 4.93 | 5.33 | 4.77 |
| 243231 | 3.80 | 3.64 | 4.25 | 6.52 | 6.64 | 5.70 | 5.13 | 7.39 | 6.66 | 8.77 | 2.31 |
| 243232 | 5.30 | 5.06 | 4.93 | 3.42 | 2.77 | 2.80 | 1.51 | 3.57 | 3.69 | 5.28 | 3.28 |
| 243286 |  | 1.39 | 1.44 | 6.89 | 6.56 | 5.92 | 6.04 | 7.47 | 6.05 | 9.07 | 4.30 |
| 243287 |  |  | 1.26 | 6.47 | 6.56 | 6.19 | 5.96 | 7.08 | 5.73 | 8.76 | 4.16 |
| 243288 |  |  |  | 6.48 | 6.03 | 5.76 | 5.84 | 6.90 | 5.31 | 8.42 | 4.47 |
| 243289 |  |  |  |  | 3.74 | 5.31 | 4.06 | 3.49 | 4.67 | 5.22 | 5.21 |
| 243568 |  |  |  |  |  | 3.28 | 2.81 | 2.98 | 3.48 | 4.06 | 5.12 |
| 243571 |  |  |  |  |  |  | 2.47 | 4.77 | 4.28 | 5.64 | 4.18 |
| 243572 |  |  |  |  |  |  |  | 3.39 | 4.24 | 5.22 | 3.56 |
| 243597 |  |  |  |  |  |  |  |  | 3.40 | 3.55 | 5.81 |
| 243598 |  |  |  |  |  |  |  |  |  | 3.76 | 5.31 |
| 243599 |  |  |  |  |  |  |  |  |  |  | 7.06 |
